# 

**Published:** 1843-10-14

**Authors:** 


☞ TO SUBSCRIBERS.—The attention of our subscribers, who are in arrears, is respectfully invited to the terms of subscription. An early attention to our claims is solicited. When it is considered that the Examiner is purely a professional journal, and unsupported by the capital of a publishing house, the propriety of a compliance with our demands will be generally recognized. Funds current in the state where the subscriber resides, will be received.

In answer to one or two complaints of irregularities or delays which have reached us, we can only state that, during the present year, the publication of this journal has been invariably punctual to the day, and that any irregularity which may have occurred is to be attributed to the Post Office department.

☞ Published every alternate Saturday, and subscriptions received, by J. C. TURNPENNY, N. E. corner of Spruce and Tenth streets. Price, Three Dollars a year, invariably in advance. Two copies sent to the same address, Five Dollars.

Communications and Letters to be addressed (always pre-paid) to the Editor of the Medical Examiner, Philadelphia.

Postmaster's franks can always be obtained for remittances.

This journal is subject to newspaper postage only.

MERRIHEW AND THOMPSON, PRINTERS, 7 CARTER'S ALLEY.

